# Variation of prevalence of malaria, parasite density and the multiplicity of *Plasmodium falciparum* infection throughout the year at three different health centers in Brazzaville, Republic of Congo

**DOI:** 10.1186/s12879-020-4913-3

**Published:** 2020-03-04

**Authors:** Pembe Issamou Mayengue, Dezi Kouhounina Batsimba, Roch Fabien Niama, Reyna Ibara Ottia, Alida Malonga-Massanga, Grâce Petula Urielle Fila-Fila, Gabriel Ahombo, Simon Charles Kobawila, Henri Joseph Parra

**Affiliations:** 1 Faculté des Sciences et Techniques, Université Marien Ngouabi, Brazzaville, BP 69 République du Congo; 2 Laboratoire National de Santé Publique, Brazzaville, BP 120 République du Congo; 3 Centre de Recherche et d’Initiation des Projets de Technologie, Brazzaville, BP 2499 République du Congo

**Keywords:** Uncomplicated malaria, Seasonal variation, Brazzaville, Republic of Congo

## Abstract

**Background:**

In the Republic of Congo, hot temperature and seasons distortions observed may impact the development of malaria parasites. We investigate the variation of malaria cases, parasite density and the multiplicity of *Plasmodium falciparum* infection throughout the year in Brazzaville.

**Methods:**

From May 2015 to May 2016, suspected patients with uncomplicated malaria were enrolled at the Hôpital de Mfilou, CSI « Maman Mboualé», and the Laboratoire National de Santé Publique. For each patient, thick blood was examined and parasite density was calculated. After DNA isolation, MSP1 and MSP2 genes were genotyped.

**Results:**

A total of 416, 259 and 131 patients with suspected malaria were enrolled at the CSI «Maman Mboualé», Hôpital de Mfilou and the Laboratoire National de Santé Publique respectively. Proportion of malaria cases and geometric mean parasite density were higher at the CSI «Maman Mboualé» compared to over sites *(P-value* <0.001). However the multiplicity of infection was higher at the Hôpital de Mfilou (*P-value* <0.001).

At the Laboratoire National de Santé Publique, malaria cases and multiplicity of infection were not influenced by different seasons. However, variation of the mean parasite density was statistically significant (*P-value* <0.01).

Higher proportions of malaria cases were found at the end of main rainy season either the beginning of the main dry season at the Hôpital de Mfilou and the CSI «Maman Mboualé»; while, lowest proportions were observed in September and January and in September and March respectively. Higher mean parasite densities were found at the end of rainy seasons with persistence at the beginning of dry seasons. The lowest mean parasite densities were found during dry seasons, with persistence at the beginning of rainy seasons. Fluctuation of the multiplicity of infection throughout the year was observed without significance between seasons.

**Conclusion:**

The current study suggests that malaria transmission is still variable between the north and south parts of Brazzaville. Seasonal fluctuations of malaria cases and mean parasite densities were observed with some extension to different seasons. Thus, both meteorological and entomological studies are needed to update the season’s periods as well as malaria transmission intensity in Brazzaville.

## Introduction

Malaria is still one of the major health problems worldwide. The global incidence of the disease in 2017 has been estimated at 219 million of cases with 435,000 deaths [1]. The sub-Saharan Africa continues to experience considerable burden of the disease with approximately 92% of malaria cases and 93% of deaths occurring in the World Health Organization (WHO) African Region [1].

Climate change has been noticed worldwide with impact on rainfall, temperature and humidity; three factors that are known to affect malaria seasonality as well as, transmission intensity [2–4]. Several studies have demonstrated the influence of these factors on the development of malaria parasites in the mosquitos [4–9] with immediate consequences on the parasite transmission to human host.

In areas with seasonal and intense malaria transmission, the human parasite reservoir declines through the dry season until the beginning of the wet season at which time vector numbers begin to rise [10]. Thus understanding impact of seasonal variation on parasite prevalence is relevant for improvement of intervention strategies towards prevention and elimination.

Entomological surveys are encouraged for this purpose. However, parasitological data such as parasite density should be able to supplement entomological data for better understanding of local seasonality and heterogeneity of exposure [11].

To predict the effect of intervention outcomes in seasonal malaria settings, it is also necessary to understand the dynamic of natural acquired immunity or premunition across a seasonal time scale [11]. The multiplicity of *Plasmodium falciparum* infection (MOI), defined as the minimum number of *Plasmodium falciparum* genotypes per infected subject, is thought to be a useful parasitological indicator of transmission or host acquired immunity level [12], and to influence the risk of subsequent malaria attacks [13]. However several studies have shown an inverse association between MOI and malaria attacks [14, 15], while others have shown the positive correlation between MOI and clinical *Plasmodium falciparum* infection [16, 17].

In the Republic of Congo, malaria is still the leading cause of attendance in health facilities with 52, 8% outpatient consultation, 44, 1% hospitalization and 28% of deaths due to malaria; and the most vulnerable are pregnant women and children under 5 years old [18].

A study conducted in 2006 in Republic of Congo jointly by the WHO and the Ministry of Health and Population showed that the transmission dynamic of malaria in the country follows two different patterns: (1) a year-round perennial transmission in forest areas, with an estimated entomological inoculation rate (EIR) of 200–1000 infective bite/person/year, and (2) a seasonal transmission in savanna areas where the high transmission period lasts 7–10 months and is directly correlated with the rainfall and the EIR is estimated to be 80–200 infective bites/ person/year [19]. In these last years, hot temperature and seasons distortions which may influence malaria seasonality have been observed in the Republic of Congo. Thus, to better control malaria intervention by predicting the optimal times at which to deploy vector control and drug-based interventions in this area in the perspective of malaria elimination, the actual profile of malaria variation is needed.

This study investigated the seasonality of *Plasmodium falciparum* malaria cases, parasite density and the MOI in Brazzaville, the Republic of Congo.

## Methods

### Study areas

The study was conducted in Brazzaville, the political capital hosting 38% (1,642,105 inhabitants) of the total population of the Republic of Congo**,** estimated at 4312715 inhabitants as described elsewhere [20, 21]. Due to the fluctuation of malaria transmission in Brazzaville, which varies from low, moderate to intense with meso-, hyper- to perennial endemicity, three different centers were considered for patients recruitment: Centre de Santé Intégré (CSI) « Maman Mboualé» located in the north part of city (4°13′S, 15°17′E); Hôpital de Mfilou located in the south part of the city (4°15′S, 15°13′E) and the Laboratoire National de Santé Publique (LNSP) located in the center part of city (4°16′S, 15°15′E). Instead of their location, the malaria transmission variation, the CSI « Maman Mboualé» and Hôpital de Mfilou have been also selected based and their ability to receive many patients from all socio-economic status and the LNSP is the national reference laboratory as described elsewhere [20, 21].

Malaria infection is primarily due to *Plasmodium falciparum* and *Anopheles gambiae s.s.* is the predominant vector. Two rainy seasons are observed each year with the main one during the months of February to May, and a short one from October to November [22–24]. The dry seasons are from June to September and from December to January.

### Study population, blood samples and data collection

From May 2015 to May 2016, patients with clinical signs of uncomplicated malaria, presenting at the laboratory at each of the three study sites were invited to participate in this study. Exclusion criteria were pregnancy, severe malaria or other severe illness as judged by the attending physician. The number of representative patients to be included in each site was estimated taking into account the proportion of malaria reported in each health center, 1 year before starting the study as described elsewhere [20, 21]. Thus, 310, 200 and 100 were a minimum number of patients to be recruited at the CSI « Maman Mboualé», Hôpital de Mfilou and the LNSP, respectively. After informed consent was obtained, records were made on patient demographics, fever or history of fever in the last 48 h, other signs of malaria, provenance, previous antimalarial drugs intake, and insecticide treated nets. The axillary temperature was taken for fever confirmation. At each study site, two thick blood smears were prepared for each patient, with one being read immediately to inform the patient of the respective result. Finger prick blood from each patient was blotted on the Whatman filter paper (3MM CHR) while preparing the thick blood smears, dried and transferred to the LNSP, where isolation of deoxyribonucleic acid (DNA) and polymerase chain reaction (PCR) were performed. Before reading, thick blood smears were dried and stained with 10% Giemsa solution (Sigma Chemical, Sigma Aldrich ChemieGmbh, Taufkirchen, Germany) in pH 7.2, for approximately 10 min. The stain was gently washed away by adding drops of clean water and the slide was completely dried before examination. Thick blood smears were assessed by experienced micrsocopists until 200 leucocytes had been counted. Parasite density was calculated for each patient assuming an average of 8000 leucocytes per μl of blood using the proposed method of the WHO [25]. Individual diagnostic result was given to each patient and advised to meet the prescribers for possible antimalarial chemotherapy. The second unstained thick blood smear was transferred to the Centre Hospitalier Universitaire de Brazzaville, a bigger referral hospital with a reference laboratory in Brazzaville for microscopy quality control as described by Mayengue et al. [20].

### Extraction of parasite DNA

Genomic DNA was extracted from samples collected on the Watman filter paper using QIAamp DNA mini Kit (Qiagen, Hilden, Germany) according to the manufacturer’s instruction. Extracted DNA was stored at − 20 °C until use.

### Parasites genotyping

*Plasmodium falciparum* genotyping was performed using the nested PCRs technique. The MSP1 and MSP2 genes in their highly polymorphic loci, namely MSP1 block 2 and MSP2 central region were used as markers for this genotyping as described previously [26, 27]. PCR amplification was performed following a 2-step amplification procedure, in which the initial amplifications were followed by individual nested PCR reactions using specific primers for K1, Mad20 and RO33 allelic families for MSP1, and FC27 and 3D7 allelic families for MSP2. Allelic and DNA free negative controls were included in each step of the reaction. Five microliters of each of the PCR products were loaded on 2% agarose gel (PeqLab, Erlangen, Germany), stained with ethidium bromide, separated by electrophoresis and visualized under ultraviolet trans-illumination. The number of products, corresponding to number of infecting MAD20, RO33 and K1 clones for MSP1 gene as well as FC27 and 3D7 clones for MSP2 gene was counted after visualization.

### Data analysis

Data collected were summarized with average and standard deviation for age and compared using Student’s t-test for two independent samples or the Bonferroni method for one-way analysis of variance (ANOVA) for multiple comparisons. Categorical responses were expressed as a percentage, and comparisons were made using Pearson’s χ2 test (or Fisher’s exact test if appropriate). The effect of seasonality on malaria infection was assessed using logistic regression method but no relationship was found, because some subgroups had few patients with malaria. So, the non-parametric Kruskal-Wallis test was used for determining the differences in prevalence of malaria infection between seasons. All analyses were done with SPSS statistical software for Windows, Version 24.0 (Chicago: SPSS Inc.). All tests were two tailed and *P-values* ≤ 0.05 were considered as statistically significant.

## Results

### Sociodemographic and clinical characteristics of patients

A total of 416, 259 and 131 patients with suspected malaria were enrolled at the CSI «Maman Mboualé», Hôpital de Mfilou and the LNSP respectively. Sociodemographic as well as clinical characteristics of these patients are summarized in Table 1.

**Table 1:** Characteristics of patients

Characteristics	CSI « Maman Mboualé»	Hôpital de Mfilou	LNSP	*P-value*
Total number	416	259	131	
Gender (F/M)	207/203	131/126	68/63	
Groups of age (n, %)				
<5 years	99 (23.8)	38 (14.8)	0 (0.0)	
≥ 5 years	317 (76.2)	219 (85.2)	131 (100.0)	
Mean age ± ET	14.5 ± 13.49	28.6 ± 19.82	42.31 ± 14.84	
Malaria cases (n, %)	173 (41.6)	62 (23.9)	12 (9.2)	<0.001
Geometric mean parasite density (Min-Max)	2399.30 (16–164,800)	1905.10 (16–100,000)	189.46 (16–8720)	<0.001
Multiplicity of infection				
MSP1	1.70	2.05	1.13	<0.001
MSP2	1.33	1.51	1.00	<0.001
MSP1 + 2	2.55	3.23	1.89	<0.001

Out of 259 patients enrolled at the Hôpital de Mfilou, gender and age were recorded for 257 of them, while with regards to the CSI « Maman Mboualé», out of the 416 recruited patients, 410 had records on gender. Both proportion of malaria cases and geometric mean parasite density were higher at the CSI «Maman Mboualé» compared to other sites *(P-value* <0.001).

### Relationship between the proportion of malaria cases, age and gender

All malaria patients recruited at the LNSP were more than 5 years old. While the proportion of malaria cases was not influenced by age at the Hôpital de Mfilou (Table 2), patients with the age greater than 5 years old were more infected compared to those with the less than 5 years at the CSI « Maman Mboualé» (*P-value* <0.001). With regards to gender, males were more infected than females at the Hôpital de Mfilou (*P-value* <0.012).

**Table 2:** Relationship between the proportion of malaria cases, age and gender

Center	Characteristics	All (%)	Proportion of malaria cases (%)	*P-value*
Hôpital de Mfilou	Age groups			
	< 5 years	38 (14.8)	11 (28.9)	0.413
	≥ 5 years	219 (85.2)	50 (22.8)	
	Gender			
	Female	131 (51.0)	22 (16.8)	
	Male	126 (49.0)	38 (30.2)	0.012
CSI « Maman Mboualé»	Age groups			
	< 5 years	99 (23.8)	27 (27.3)	0.001
	≥ 5 years	317 (76.2)	146 (46.1)	
	Gender			
	Female	207 (50.5)	90 (43.5)	0.549
	Male	203 (49.5)	82 (40.4)	
LNSP	Age groups			
	< 5 years	0 (0.0)	0 (0.0)	–
	≥ 5 years	131 (100)	12 (09.2)	
	Gender			
	Female	68 (51.9)	06 (08.8)	0.890
	Male	63 (48.1)	06 (09.5)	

### Genetic diversity of *Plasmodium falciparum* and relationship between the multiplicity of infection, parasite density, age and gender

The distribution of K1, Mad20 and RO33 allelic families showed the presence of 14, 17and 5 MSP1 alleles in clinical isolates from patients from Hôpital de Mfilou, CSI « Maman Mboualé», and LNSP respectively, with no statistical significant predominance of any specific family between sites (*P-value* <0.417). With regards to MSP2 gene, a total of 25, 27 and 4 different alleles of FC27 and 3D7 were identified at the Hôpital de Mfilou, the CSI « Maman Mboualé», and the LNSP respectively, with no statistical significant predominance of any specific family between sites (*P-value* <0.2862) (Table 3).

**Table 3:** Distribution of *msp-1* and *msp-2* detected allelic families according to the study sites

Center	MSP1 gene n(%)			MSP2 gene n(%)	
	K1	Mad20	RO33	FC27	3D7
Hôpital de Mfilou	51 (43.6)	33 (28.2)	33 (28.2)	41 (49.4)	42 (50.6)
CSI «Maman Mboualé»	95 (41.5)	87 (38.0)	47 (20.5)	94 (47.7)	103 (52.3)
LNSP	04 (44.4)	04 (44.4)	01 (11.2)	02 (25.5)	06 (75.0)
*P.value*	0.417			0.2862	

Regardless of the molecular marker used, the MOI was higher at the Hôpital de Mfilou compared to the CSI « Maman Mboualé», and the LNSP (Table 1), with the overall MOI of 3.23, 2.55 and 1.89 respectively (*P-value* <0.001). While the MOI was not influenced by age, patients with less than 5 years had significantly higher parasite densities compare to those with the age greater than 5 years old (Table 4). In addition, parasite densities were not influenced by gender regardless of the site, while males had higher MOI compared to females at the Hôpital de Mfilou. Moreover, the MOI was not associated with parasite density.

**Table 4:** Relationship between the multiplicity of infection, parasite density and age

Center	Characteristics	Mean parasite densities	*P-value*	MOI	*P-value*
Hôpital de Mfilou	Age groups				
	< 5 years	22,167.18	0.037	3.25	0.956
	≥ 5 years	9847		3.15	
	Gender				
	Female	20,092.53	0.197	1.88	0.007
	Male	10,161.76		2.38	
CSI « Maman Mboualé»	Age groups				
	< 5 years	32,109.11	0.012	2.18	0.469
	≥ 5 years	15,816.28		2.63	
	Gender				
	Female	18,330.68	0.961	2.14	0.841
	Male	18,645.53		2.17	
LNSP	Age groups				
	< 5 years	–	–	–	–
	≥ 5 years	1320.33		1.89	
	Gender				
	Female	932.00	0.639	1.48	0.876
	Male	1708.67		1.50	

### Variation of proportion of malaria cases and parasites density throughout the year

During the year, *Plasmodium falciparum* was the only species identified in all positive slides confirmed by the quality control expert.

A particular profile has been found at the LNSP with very low malaria cases without significance between seasons (*P-value* = 0.477). However, the difference of the mean parasite density was statistically significant (*P-value* <0,01) with the highest peak in November, corresponding to the rainy season.

At the Hôpital de Mfilou, highest proportions of malaria cases have been found at the beginning of the study in May and June (50%), November (40%), February (38.9%) and April (33.3%) corresponding to the months of rainy seasons and the beginning of dry season for June (Table 5). However, lowest proportions of cases were noticed in September (3.4%), and January (18.8%) corresponding to the peak of dry seasons. The variation of these proportions within the year was statistically significant (*P-value* <0.004). A contrasting profile was observed at the CSI « Maman Mboualé», where the highest proportion of malaria cases were obtained during the main rainy season, in April, May including the beginning of dry season in June. Progressive diminution of malaria cases has been noticed from July to March, reaching the lowest proportion in December and March corresponding to the beginning of dry season and main rainy season respectively (*P-value* <0.01). When taking all tree sites together the highest proportions of malaria cases were confirmed during the rainy seasons, while lowest proportions were registered in September, December and March (*P-value* <0.01).

**Table 5:** Relationship between different seasons and proportion of malaria cases

Seasons	Months	Hôpital de Mfilou		CSI «Maman Mboualé»		LNSP		All	
		N	n (%)	N	n (%)	N	n (%)	N	n (%)
Rainy	May-15	4	2 (50,0)	8	6 (75,0)	4	0 (0,0)	16	8 (50,0)
Dry	June-15	24	12 (50,0)	36	29 (80,6)	14	1 (7,1)	74	42 (56,8)
	July-15	27	7 (25,9)	36	17 (47,2)	14	0 (0,0)	77	24 (31,2)
	August-15	32	3 (9,4)	34	15 (44,1)	5	0 (0,0)	71	18 (25,4)
	September-15	29	1 (3,4)	36	11 (30,6)	18	4 (22,2)	83	16 (19,3)
Rainy	October-15	25	6 (24,0)	34	9 (26,5)	7	0 (0,0)	66	15 (22,7)
	November-15	15	6 (40,0)	34	10 (29,4)	6	1 (16,7)	55	17 (30,9)
Dry	December-15	23	6 (26,1)	31	6 (19,4)	14	2 (14,3)	68	14 (20,6)
	January-16	16	3 (18,8)	32	10 (31,3)	11	1 (9,1)	59	14 (23,7)
Rainy	February-16	18	7 (38,9)	34	14 (41,2)	13	1 (7,7)	65	22 (33,8)
	March-16	16	2 (12,5)	37	9 (24,3)	4	1 (25,0)	57	12 (21,1)
	April-16	9	3 (33,3)	32	16 (50,0)	7	0 (0,0)	48	19 (39,6)
	May-16	21	4 (19,0)	32	21 (65,6)	14	1 (7,1)	67	26 (38,8)
*P-value*		–	0,004	–	<0,01	–	0,477	–	<0,01

N: number of enrolled patients per month; n: number of malaria cases per month

**Fig. 1:**
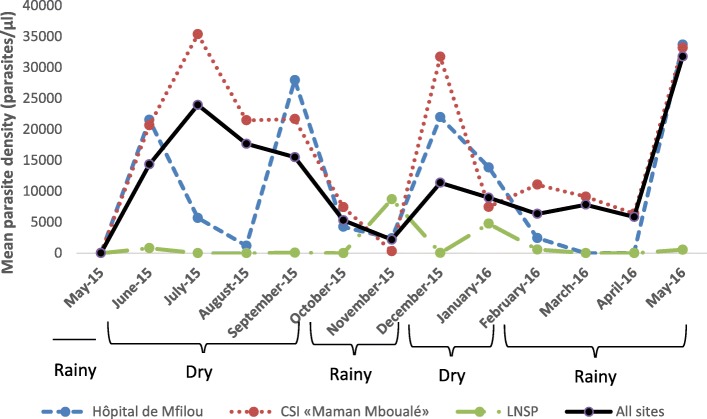
Variation of mean parasite density throughout the year. The figure shows mean asexual parasite densities calculated monthly from May 2015 to May 2016 at each study site and when taking all three sites together. Different months were grouped into dry and rainy seasons

With regards to mean asexual parasite densities, a clear seasonality has been noticed at the Hôpital de Mfilou, with the highest peaks mainly observed at the beginning of dry seasons in June and December as well as in May (corresponding to the end of rainy season) at the end of the study (Fig. 1). However the only malaria case registered in September had also a high parasite density. Moreover, it is obvious from the result that the periods of low mean asexual parasite densities were observed at the peak of the dry season corresponding to the month of August with persistence during rainy seasons (Fig. 1) corresponding to October and November as well as, February March and April for the short and main rainy seasons, respectively.

By considering the CSI «Maman Mboualé», tree high peaks of mean parasite density have been identified in July, December and May; thereafter, persistence decrease has been noticed during the dry season, including the rainy seasons (Fig. 1) corresponding to October and November as well as from February to April (*P-value* <0.043). When taking all tree sites together, similar profile with the CSI «Maman Mboualé», has been observed (*P-value* <0.01).

### Variation of multiplicity of *Plasmodium falciparum* infection throughout the year

Particular profile has also been found at the LNSP regarding the MOI with no infection in some alternative months (May, July, August, October and April), and the MOI did not vary significantly over the year (*P-value* = 0,853).

**Fig. 2:**
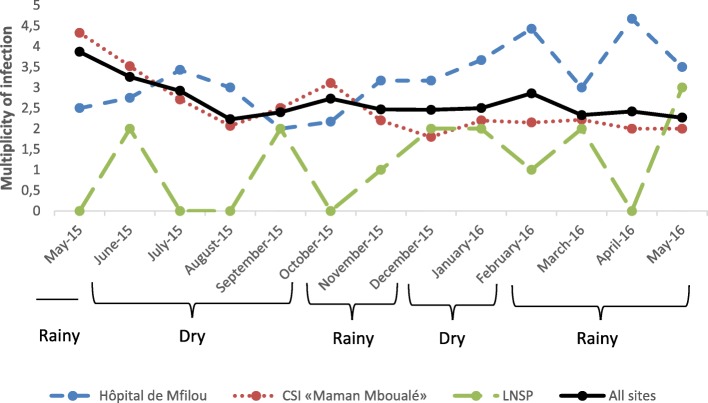
Variation of multiplicity of *Plasmodium falciparum* infection throughout the year. The figure shows multiplicity of *Plasmodium falciparum* infection determined monthly from May 2015 to May 2016 at each study site and when taking all three sites together. Different months were grouped into dry and rainy seasons

At the Hôpital de Mfilou, significant variation of MOI was found over the year (*P-value* <0.01) without any clear pattern and devoid of seasonality. From the beginning of the study in May, the MOI increased reaching a peak during the main dry season in July, thereafter persistence of decrease was observed, with the lowest MOI in September corresponding to the end of the main dry season (Fig. 2). There was a permanent increase of MOI from October, reaching the highest peaks in February and April, regardless the short dry season in December and January.

Concerning the CSI «Maman Mboualé», tree different peaks were observed, with highest MOI registered at the beginning of the study in May; follow by those in October and the lowest in December. Significant variation of MOI was observed within the main dry, short rainy and dry seasons, while MOI was slightly stable during the main rainy season from February to May (*P-value* <0.01). Persistence of decrease of MOI was observed from June to August and an increase in September during the main dry season, while high peak of MOI was observed in October with a decrease in November during the short rainy season. Inversely, lower peak was observed in December with an increase in January during the short dry season. Thus there was no clear pattern and devoid of seasonality. When taking all tree sites together, significant variation of MOI was also observed over the year (*P-value* <0.01) with similar profile with the CSI «Maman Mboualé» from May at the beginning of the study to November, but slightly increase of MOI from December to May at the end of the study.

## Discussion

The understanding of malaria dynamic in the area is crucial by targeting the peak malaria transmission for malaria control intervention at both vector and drug-based level. In the Republic of Congo, hot temperature and seasons distortions have been observed, shifting obviously the beginning and the end periods of different seasons, with impact on rainfall, temperature and humidity. Thus, it is urgent to evaluate malaria seasonality as well as, transmission intensity. To our knowledge, this is a first study to evaluate seasonality of the malaria parasitaemia and the MOI over the year in Brazzaville.

With regards to the variability of malaria transmission level in the different parts of Brazzaville [19], three different health facilities according to their location were considered, notably the CSI «Maman Mboualé» in the north, the LNSP in the center and the Hôpital de Mfilou in the south of Brazzaville.

The results indicate a particular profile at the LNSP with very low proportion of malaria cases being always in adults, low mean parasite density, low genetic diversity of *Plasmodium falciparum* as well as low MOI. No influence of gender on the proportion of malaria cases, the mean parasite density, the MOI as well as no influence of seasons on the MOI and the proportion of malaria cases was found at this study site. Although, the variability of the mean parasite density was noticed over the year with the highest peak in November, it is obviously difficult to draw any conclusion due to small number of malaria cases. In addition, the majority of patients recruited at the LNSP came from the distant districts of the LNSP. Thus the type of transmission as well as the impact of seasons on malaria should be discussed with caution while low level of malaria transmission was expected at this site which is more urbanized.

Proportion of malaria cases and the mean parasite density were higher at the CSI «Maman Mboualé» compared to the Hôpital de Mfilou. In addition patients with the age higher than 5 years were more likely to be infected at the CSI «Maman Mboualé» compared to the Hôpital de Mfilou. Inversely, the MOI has been found to be high at the Hôpital de Mfilou compared to the CSI «Maman Mboualé», while genetic diversity of *Plasmodium falciparum* was found, with no statistical significant predominance of any specific family between these two sites. Despite the lack of recent entomological data from Brazzaville, the number of clones coinfecting a single host can be used as an indicator of the level of malaria transmission or the level of host acquired immunity [12]. Therefore, the discrepancies on the MOI may suggest the different level of malaria transmission between the north and the south parts of Brazzaville; with CSI «Maman Mboualé» being more urbanized compared to the Hôpital de Mfilou. With regards to gender, males were likely to be more infected with high MOI compared to females at the Hôpital de Mfilou. This result may suggest the high level of exposure of males to *Plasmodium falciparum* infection.

While parasite densities were influenced by age in these two sites, the MOI was influenced neither by age nor by parasite density regardless the study site, concordant with the studies conducted in Brazzaville and Pointe Noire [28, 29]. Therefore, regardless of the parasite densities, and the fact that the sample collection was done from symptomatic infection, the prevalence of multi clonal infections affected all the two age groups.

Both proportion of malaria cases and mean parasite density were influenced by the seasonality in these two study sites, but with some particularities. The higher proportions of malaria cases were found mainly at the end of main rainy season including the month of June (which is the beginning of the main dry season). While, clear impact of dry season has been observed at the Hôpital de Mfilou with lowest proportions in September and January, at the CSI «Maman Mboualé», lowest proportions of malaria cases were found in September and March. Higher mean parasite densities were found meanly at the end of rainy seasons with persistence at the beginning of dry seasons. Inversely to the lowest mean parasite densities founded during dry seasons but with persistence at the beginning of rainy seasons. This could be due to environmental particularities including the humidity relative to the presence of swampy areas around the Hôpital de Mfilou as well as the “Fleuve Congo” river, surrounding the CSI «Maman Mboualé», which may maintain the multiplication of mosquitos until the beginning of dry seasons, while low level of humidity may influence mosquitos multiplication at the beginning of the rainy season. The outcome of this study is in agreement with those in Nigeria [30–32]. Additionally, persistence of high proportions of malaria cases and mean parasite densities at the beginning of dry seasons and their lower values at the beginning of rainy seasons may also be due to season distortions observe in Brazzaville. It has been suggested that weather variation may diminished malaria seasonality [4]. With regards to the MOI, regardless of fluctuation, decrease of MOI was observed at the CSI «Maman Mboualé» throughout the year, which may be related to the influence of weather variation on malaria transmission intensity. Thus, both meteorological data and entomological studies are needed to update the season’s periods as well as malaria transmission intensity.

Fluctuation of the MOI throughout the year was observed without any clear pattern and devoid of seasonality at the Hôpital de Mfilou and the CSI «Maman Mboualé». The findings presented in this study disagree with the results of previous study in Senegal and Ghana [13, 33]. Therefore, the discrepancies may be due to the difference of population groups with the current study being conducted in symptomatic population. Further studies are needed including asymptomatic population to better evaluate the impact of seasonality on the MOI in the Republic of Congo. Interestingly, alternated fluctuation of MOI was observed between these two study sites throughout the year. This observation supports the different level of malaria transmission which may exist between the north and the south parts of Brazzaville.

## Conclusion

With the lack of recent entomological data in Brazzaville, this study conducted throughout the year on *Plasmodium falciparum* symptomatic population suggests that malaria transmission is still variable between the north and the south of Brazzaville. Seasonal fluctuation of proportion of malaria cases and mean parasite density was observed. However, persistence of high proportions of cases and mean parasite densities at the beginning of dry seasons and their lower values at the beginning of rainy seasons may be due to the season distortions observe in Brazzaville. Thus, both meteorological data and entomological studies are needed to update the season’s periods as well as malaria transmission intensity.

## Data Availability

The data generated and analyzed in this study are not publicly available for ethical reasons. However, they may be available from the corresponding author upon request.

## Abbreviations

CERSSA: Comité d’ethique de la recherche en sciences de la santé
CSI: Centre de santé intégré
DNA: Deoxyribonucleic acid
LNSP: Laboratoire national de santé publique
MOI: Multiplicity of *Plasmodium falciparum* infection
MSP: Merozoite surface proteins
PCR: Polymerase chain reaction
WHO: World health organization
